# Pulmonary embolism and 3-month outcomes in 4036 patients with venous thromboembolism and chronic obstructive pulmonary disease: data from the RIETE registry

**DOI:** 10.1186/1465-9921-14-75

**Published:** 2013-07-18

**Authors:** Laurent Bertoletti, Sara Quenet, Silvy Laporte, Joan Carles Sahuquillo, Francisco Conget, José María Pedrajas, Mar Martin, Ignacio Casado, Antonio Riera-Mestre, Manuel Monreal

**Affiliations:** 1 Thrombosis Research Group, EA3065, University Saint-Etienne, Jean Monnet, Saint-Etienne F-42023, France; 2 CIE3, INSERM, Saint-Etienne F- 42055, France; 3 Department of Therapeutic Medicine, CHU Saint-Etienne, Hôpital Nord, Saint-Etienne F-42055, France; 4 Department of Internal Medicine, Hospital Municipal de Badalona, Barcelona, Spain; 5 Department of Pneumonology, Hospital Clínico Universitario Lozano Blesa, Zaragoza, Spain; 6 Department of Internal Medicine, Hospital Clínico San Carlos, Madrid, Spain; 7 Department of Internal Medicine, Hospital Infanta Sofía, Madrid, Spain; 8 Department of Pneumonology, Hospital Universitario Virgen de las Nieves, Granada, Spain; 9 Department of Internal Medicine, Hospital Univesitari de Bellvitge - IDIBELL, Barcelona, Spain; 10 Department of Internal Medicine, Hospital Universitari Germans Trias I Pujol, Badalona, Spain

**Keywords:** Chronic obstructive pulmonary diseases, Deep venous thrombosis, Prognosis, Pulmonary embolism, Venous thromboembolism

## Abstract

**Background:**

Patients with chronic obstructive pulmonary disease (COPD) have a modified clinical presentation of venous thromboembolism (VTE) but also a worse prognosis than non-COPD patients with VTE. As it may induce therapeutic modifications, we evaluated the influence of the initial VTE presentation on the 3-month outcomes in COPD patients.

**Methods:**

COPD patients included in the on-going world-wide RIETE Registry were studied. The rate of pulmonary embolism (PE), major bleeding and death during the first 3 months in COPD patients were compared according to their initial clinical presentation (acute PE or deep vein thrombosis (DVT)).

**Results:**

Of the 4036 COPD patients included, 2452 (61%; 95% CI: 59.2-62.3) initially presented with PE. PE as the first VTE recurrence occurred in 116 patients, major bleeding in 101 patients and mortality in 443 patients (Fatal PE: first cause of death). Multivariate analysis confirmed that presenting with PE was associated with higher risk of VTE recurrence as PE (OR, 2.04; 95% CI: 1.11-3.72) and higher risk of fatal PE (OR, 7.77; 95% CI: 2.92-15.7).

**Conclusions:**

COPD patients presenting with PE have an increased risk for PE recurrences and fatal PE compared with those presenting with DVT alone. More efficient therapy is needed in this subtype of patients.

## Background

Chronic obstructive pulmonary disease (COPD) is a common and severe medical condition: it affects more than 10% of people over 40 years old [1] and the World Health Organization estimates it will become the fourth leading cause of death in 2030 worldwide [2]. Recent data showed that about one in every four individuals will be diagnosed for COPD during their lifetime [3].

COPD is recognized as a moderate risk factor for another frequent disease : venous thromboembolism (VTE), in the same group than cancer or hormonal therapy [4]. Pulmonary embolism (PE) and deep venous thrombosis (DVT) are the two clinical presentation forms of VTE. DVT is two times more frequent than PE in the general setting, whereas PE have a higher risk of death [5]. As patients with COPD have reduced pulmonary vascular reserve, PE is considered to be a major threat in COPD patients, reported to be responsible of at least 10% of deaths [6] but its evocation may be challenging, particularly during an acute exacerbation of COPD [7-9].

We recently found that COPD patients with VTE have a higher embolic “tropism” than non COPD patients [10]. The clinical presentation of VTE (say PE or DVT) in COPD patients is modified with higher PE/DVT ratio than non COPD patients. They also experiment higher rates of VTE recurrences as PE, and higher rates of fatal PE. In the general setting, these outcomes are influenced by the initial VTE clinical presentation (say PE or DVT). For example, patients presenting initially with DVT have higher risk to present with DVT rather than PE in case of recurrence. It is unclear if it remains true in COPD, because of its higher embolic tropism. Moreover, these worse outcomes might prompt clinicians to increase the antithrombotic pressure, but we also found that COPD patients experiment higher risk of bleeding than non-COPD patients and this point should conversely prompt clinicians to avoid exposure to an increase risk of bleeding.

Hence, we aimed to assess the association between the clinical presentation of VTE (PE or DVT) and the risk of 3-month adverse outcomes (i.e. PE, major bleeding and death during the follow-up) in patients with an objectively confirmed acute symptomatic VTE and underlying COPD from the RIETE Registry.

## Materials and methods

### Registry design

The RIETE registry is an ongoing, international, multicenter, prospective cohort of consecutive patients with symptomatic, objectively confirmed, acute VTE (DVT, PE, or both). Patients are managed according to the clinical practice of each participating hospital center. The only exclusion criterium is participation in a therapeutic clinical trial with blind medication. Demographic data and comobidities such as cancer, COPD, chronic heart failure or renal insufficiency at the time of the index event are systematically prospectively recorded by the treating physician. For this analysis, only COPD patients were considered. At each participating center, a registry coordinator controlled the quality of data collection (eg, internal validity and coherence) and recorded the data from each patient on a computer-based case report form. Coordinators ensured that all consecutive patients with confirmed VTE were included in the registry. In addition, the database of each analysis was controlled. The information was then transferred online via a secure Web site to the Study Coordinating Centre responsible for data management. Data quality was also monitored by members of contract research organizations who compared the data on medical records with the data transferred online during periodic visits to participating hospitals. All patients provided oral or written consent to their participation in the registry, in accordance with the requirements of the ethics committee of each country.

### Study variables and definition

For the purpose of the present study, COPD patients were divided according to their initial VTE presentation: PE patients defined as patients with an objectively confirmed initial symptomatic PE (with or without DVT) or patients who died from PE less than 8 days after their first symptoms of PE with no evidence of PE recurrence, and DVT patients defined as patients with an objectively confirmed initial symptomatic DVT without symptomatic PE. The following information was also collected: demographic data, symptoms on presentation, types and results of VTE diagnosis methods, risk factors for VTE, and the 3-month outcomes. Recent immobilization is defined as an immobilization for non-surgical reason ≥4 days in the 2-month period prior to VTE diagnosis. Recent surgery is defined as operation in the 2 month prior to VTE. Obesity is defined by a BMI>30 kg/m^2^.

### Study outcomes

In the present study, the occurrence of an objectively confirmed PE, major bleeding, fatal PE and all-cause death were the outcomes of interest that were analyzed during a 3-month follow-up period. In patients with acute respiratory symptoms suggesting PE, symptomatic PE was confirmed if it was documented objectively (positive helical computed tomography scan, high-probability ventilation–perfusion lung scan, positive pulmonary angiography, visualization of thrombus in right ventricle or right atrium on echocardiography, or intermediate-probability ventilation–perfusion lung scan associated with deep-vein thrombosis in the lower limbs confirmed by compression ultrasonography or contrast venography). If the patient died, death was considered to be due to PE if this diagnosis had been documented at autopsy, or if the patient died shortly (less than 10 days) after objectively confirmed symptomatic PE, and in the absence of any alternative diagnosis.

Bleeding complications were classified as ‘major’ if they were overt and required a transfusion of 2 or more units of bloods, were retroperitoneal, spinal or intracranial, or were fatal.

These events were adjudicated by the RIETE registry coordinators.

### Data analysis

Differences in the distribution of characteristics between PE patients and DVT patients were assessed using Chi square tests for categorical variables and *t* test for continuous variables. Cumulative incidence rates of first VTE recurrence as PE, fatal PE (either the first or subsequent VTE recurrence), all-cause death and major bleedings were estimated using the Kaplan-Meier method and compared between PE patients and DVT patients by the log-rank test. Cox proportional hazards regression models were used to examine whether initial presentation with PE was associated with the study outcomes. Odds ratios and 95% confidence intervals (CIs) were used to quantify the associations.

Data were processed and analyzed using SAS-Windows™ software (version 9.2).

## Results

Between March 2001 and June 2011, 36949 consecutive patients over 18 years with acute, symptomatic, objectively confirmed VTE have been enrolled in RIETE. Of them, 4036 (10.9%) had COPD: 2693 patients (66.7%) were male and 1343 female. The age of the COPD population (mean±SD) was 72.85 ± 11.53 years. Cancer was reported in 888 (22.0%) of COPD patients.

### Initial VTE presentation

At study entry, 2452 COPD patients (61%) were objectively diagnosed as having PE. Compared to the 1584 DVT patients, PE patients were significantly older (patients over 75: 54.3% vs 45.9%, p<0.0001) and were more likely female (35.6% vs 29.7%, p<0.0001). Conversely, obesity as well as a history of VTE were less often associated with PE at presentation (respectively 19.2% vs 22.5%, p=0.02; 15.6% vs 18.3%, p=0.02) (Table 1).

**Table 1:** Baseline characteristics of 4036 patients with COPD according to initial VTE presentation

	**PE patients**	**DVT patients**	**p**
	**(n=2452), %**	**(n=1584), %**	
Men	1579 (64.4)	1114 (70.3)	<0.0001
Obesity (BMI > 30 kg/m^2^)	470 (19.2)	356 (22.5)	0.02
Age			
Mean (SD)	73.4 (11.4)	72.0 (11.7)	<0.0001
≥75 years	1332 (54.3)	727 (45.9)	<0.0001
Cancer	535 (21.8)	353 (22.3)	0.73
Recent surgery	197 (8.0)	111 (7.0)	0.23
Previous VTE	383 (15.6)	290 (18.3)	0.02
Recent immobilization > 3 days	780 (31.8)	491 (31.0)	0.59

Recent immobilization is defined as an immobilization for non-surgical reason ≥4 days in the 2-month period prior to VTE diagnosis. Recent surgery is defined as operation in the 2 month prior to VTE.

### First VTE recurrence as PE during the 3-month follow-up

**Figure 1:**
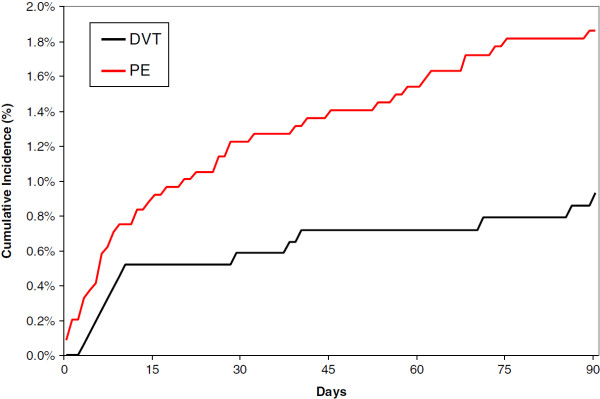
PE recurrences according to initial presentation as DVT or PE.

At 3 months, the first VTE recurrence as PE occurred in 116 patients (2.9%; 95% confidence interval (CI): 2.4-3.4) (Figure 1). The cumulative incidence rates of first symptomatic VTE recurrence as PE reaches 4.1% (95% CI : 3.4%-5.0%) in PE patients vs 1.1% (95% CI: 0.7%-1.8%) in DVT patients (p_Logrank_<0.0001) (Table 2). During this period, the cumulative incidence rates of first symptomatic VTE recurrence as DVT was 0.4% (95% CI: 0.2%-0.9%) in PE patients vs 1.5% (95% CI: 1.0%-2.1%) in DVT patients. Predictive factors associated with VTE recurrence as PE are presented in univariate (Table 3) and multivariate analysis (Table 4). The risk of a first VTE recurrence as PE was increased in patients initially presenting with PE (OR= 2.04 (1.11-3.72), p<0.01), as in patients with cancer (Table 4).

**Table 2:** Three-month cumulative incidence of study outcomes according to initial VTE presentation

	**PE patients**	**DVT patients**	***p***
	**(n=2452)**	**(n=1584)**	
First recurrent VTE as PE^a^	99 (4.1%)	17 (1.1%)	<0.0001
All-cause deaths	305 (12.5%)	138(8.7%)	0.0002
Fatal PE*	76 (3.1%)	6 (0.4%)	<0.0001
Major bleedings	70 (3.0%)	31 (2.0%)	0.08

^a^ fatal or not.

* First recurrence or subsequent fatal PE.

**Table 3:** Univariable analysis for recurrent VTE, recurrent PE and fatal PE

	**Recurrent VTE**	**Recurrent PE**	**Fatal PE**	**Major bleeding**
**Men**	68 (76%)	42 (74%)	55 (66%)	65 (66%)
	OR= 1.56 (0.96-2.53)	OR= 1.41 (0.78-2.55)	OR= 0.98 (0.62-1.56)	OR= 0.99 (0.64-1.51)
**Obesity (BMI > 30 kg/m**^**2**^**)**	24 (35%)	12 (28%)	12 (18%)	22 (36%)
(N=2728)	OR= 1.26 (0.76-2.09)	OR= 0.89 (0.45-1.74)	OR= 0.50 (0.27-0.95)	OR= 1.30 (0.77-2.20)
**Age ≥75 years**	38 (42%)	21 (37%)	53 (64%)	63 (64%)
	OR= 0.70 (0.46-1.07)	OR= 0.56 (0.33-0.96)	OR= 1.72 (1.10-2.71)	OR= 1.76 (1.16-2.68)
**Cancer**	32 (36%)	19 (33%)	32 (39%)	25 (26%)
	OR= 1.98 (1.28-3.07)	OR= 1.78 (1.02-3.11)	OR= 2.26 (1.44-3.54)	OR= 1.22 (0.77-1.93)
**Recent surgery**	7 (7.8%)	3 (5.3%)	4 (4.8%)	13 (13%)
	OR= 1.02 (0.47-2.23)	OR= 0.67 (0.21-2.15)	OR= 0.61 (0.22-1.67)	OR= 1.90 (1.05-3.44)
**Previous VTE**	15 (17%)	7 (12%)	9 (11%)	18 (18%)
	OR= 1.00 (0.57-1.75)	OR= 0.70 (0.31-1.54)	OR= 0.60 (0.30-1.21)	OR= 1.12 (0.67-1.89)
**Recent immobilization**	26 (29%)	17 (30%)	44 (53%)	39 (39%)
	OR= 0.96 (0.61-1.53)	OR= 1.01 (0.57-1.79)	OR= 2.75 (1.77-4.25)	OR= 1.59 (1.06-2.40)
**Initial presentation as PE**	53 (59%)	43 (75%)	76 (92%)	68 (69%)
	OR= 0.91 (0.60-1.39)	OR= 1.97 (1.08-3.62)	OR= 7.10 (3.27-15.4)	OR= 1.46 (0.94-2.25)

**Table 4:** Multivariable analysis for recurrent VTE, recurrent PE, fatal PE and major bleeding

	**Recurrent VTE**	**Recurrent PE**	**Fatal PE**	**Major bleeding**
Cancer	2.11 (1.37-3.26)	1.91 (1.10-3.31)	3.43 (2.11-5.59)	-
	p-value=0.001	p-value=0.022	p-value=<0.001	
Recent surgery	-	-	-	2.41 (1.30-4.46)
				p-value=0.005
Recent immobilization	-	-	3.53 (2.16-5.77)	1.88 (1.23-2.88)
			p-value=<0.001	p-value=0.004
Initial presentation as PE	-	2.04 (1.11-3.72)	6.77 (2.92-15.7)	-
		p-value=0.021	p-value=<0.001	

### Major bleeding

**Figure 2:**
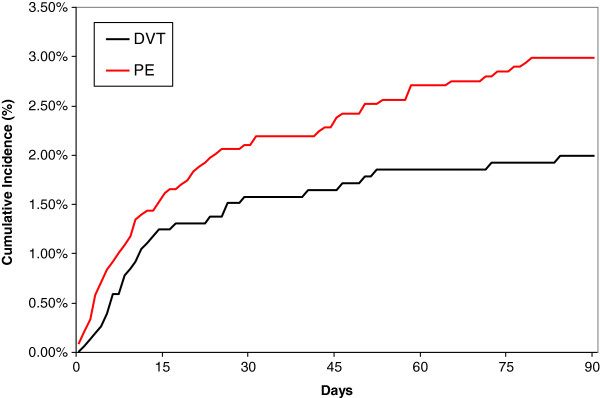
Major bleeding according to initial presentation as DVT or PE.

Three months after the initial VTE event, 101 patients (2.5%; 95% CI: 2.1-3.0) presented with a major bleeding (Figure 2). Of these, 25 (25%) died of the bleeding event. Major bleeding cumulative incidence at 3 months was 3.0% (95% CI: 2.4%-3.8%) in PE patients and 2.0% (95% CI: 1.4%-2.8%) in DVT patients (p_Logrank_=0.06) (Table 2). There was a trend toward an increased risk of major bleeding in patients presenting with PE in univariate analysis (Table 3), but it failed to reach significance (OR= 1.46 (0.94-2.25) and was no more significant in the multivariate analysis (Table 4). Only recent surgery or immobilization were significantly associated with the risk of major bleeding.

### Fatal PE and all-cause death

**Figure 3:**
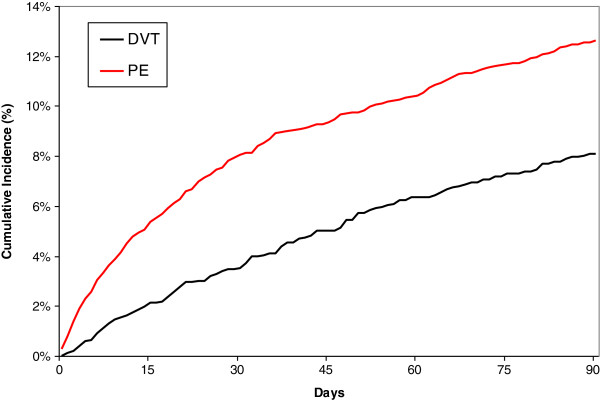
Mortality according to initial presentation as DVT or PE.

Overall, 443 (cumulated incidence of 11.0%; 95% CI: 10.0-12.0) patients died during the 3-month study period (Figure 3): 82 died of PE, 25 of bleeding, and 336 died for other reasons (Table 5). The cumulative incidence of death was higher in PE patients (12.5% (11.2%-13.8%)) compared to DVT patients (8.7% (7.4%-10.2%)) (p_Logrank_=0.0001)(Table 2).

**Table 5:** Reported causes of death, according to initial VTE presentation

	**PE patients**	**DVT patients**
	**(n=305)**	**(n=138)**
Fatal pulmonary embolism	76 (24.9%)	6 (4.3%)
Respiratory insufficiency	55 (18.0%)	22 (15.9%)
Neoplasia	44 (14.4%)	37 (26.8%)
Infection	32 (10.5%)	14 (10.1%)
Fatal bleeding	17 (5.6%)	8 (5.8%)
Heart failure	10 (3.3%)	6 (4.4%)
Sudden death	6 (2.0%)	6 (4.4%)
Multi-organ failure	6 (2.0%)	5 (3.6%)
Myocardial infarction	5 (1.6%)	2 (1.5%)
Bowel occlusion	3 (1.0%)	1 (0.7%)
Liver insufficiency	2 (0.7%)	1 (0.7%)
Stroke	1 (0.3%)	2 (1.4%)
Terminal renal insufficiency	1 (0.3%)	2 (1.4%)
Critical limb ischemia	1 (0.3%)	0
Other	11 (3.6%)	2 (1.5%)0
Unknown	35 (11.5%)	24 (17.4%)

At 3 months, fatal PE was retained for 82 patients (2.0%; 95% CI: 1.6-2.5). The cumulative incidence rates of fatal PE were 3.1% (95% CI: 2.5% - 3.9%) in PE patients vs 0.4% (95% CI: 0.2 – 0.9%) in DVT patients (p_Logrank_<0.0001) (Table 2). Predictive factors associated with Fatal PE are presented in univariate (Table 3) and multivariate analysis (Table 4). The risk of fatal PE was increased in patients initially presenting with PE (OR= 6.77 (6.77 (2.92-15.7), p<0.01), as in patients with cancer or those recently immobilized (Table 4).

## Discussion

To the best of our knowledge, the present study is the largest clinical cohort focusing on patients presenting with COPD and VTE. We found that COPD patients presenting initially with PE concentrate the worse outcomes and represent a therapeutic challenge, with more recurrent VTE as PE and Fatal PE, but also a trend-toward an increased risk of major bleeding.

In our study, 61% of COPD patients were objectively diagnosed as having PE. We have recently pointed out this modified clinical presentation [10], which may be explained by the presence of already-known condition leading to chronic dyspnea (say COPD) or by chronic systemic inflammation. Another recent work describes similar results in asthma, another pulmonary chronic inflammatory disease [11].

COPD patients initially presenting with PE were more likely to suffer recurrence as PE than DVT patients. The impact of initial clinical presentation on the form of recurrence is currently still under debate. For example, PE was the form of recurrence in half of the patients included in the EINSTEIN DVT study [12], while patients with PE were shown to be at higher risk of recurrence as PE than DVT patients in the general settings [13]. Our 3.9-times increased risk is close to the 3-times increased risk of PE in PE patients compared to DVT patients found in Baglin’s study. According to recommendations regarding at-home treatment [14], we may hypothesize that hospitalized DVT patients are at higher risk of recurrence and closer to PE patients than DVT patients managed at home. This argument is reinforced by the way we classified patients as presenting with PE. The presence of symptoms was a requisite. However, some patients in the DVT group may also have presented with an asymptomatic PE [15], leading to shrinkage of the difference between the PE recurrence risk of PE patients and DVT patients. In addition, as diagnosis of PE may be difficult in COPD patients since clinical signs of COPD mimic PE [16,17], it is possible than some recurrent PE were under-diagnosed in our study, in both groups of patients.

The risk of major bleeding was 1.5 (95% CI of 0.9 to 2.2) higher in PE patients compared to DVT patients as found in the whole VTE population from the RIETE registry [18], despite the anticoagulation target does not differ usually between DVT and PE patients [19]. The non-statistically significant results in the subgroup of COPD patients may be due to a lack of power.

The overall 3-month all-cause mortality in these patients with concomitant COPD and VTE is estimated to be 11.0%, three-time higher than in a recent randomized control trial including stable COPD patients [20] and as high as that one observed in COPD patients admitted in a respiratory intensive care unit for a severe exacerbation [21]. Besides the presence of PE in 60% of our patients, this high mortality may be explained by the characteristics of our population: elderly patients (51% over 75), 22% of cancer. In this population at high risk of death, PE as the presentation of the initial VTE increased the risk of death by 1.5 (95% CI of 1.2 to 1.8). Despite significant therapeutic improvement in the management of VTE and COPD, this result was already reported in the same proportion more than twenty years ago (when none of the COPD current treatments were available) in a small and highly-selected group of COPD patients [22] and already described in patients with VTE [23,24]. The association between PE at presentation and a higher mortality raises the possibility that some recurrent PE could have been fatal, but classified as respiratory failure secondary to a severe COPD exacerbation. Fatal PE may then have been underdiagnosed. Of note, fatal PE represents the main cause of death in patients, particularly in patients presenting with PE. In COPD initially presenting with PE the incidence of recurrent PE and major bleeding was similar (69 vs. 70 events, respectively), but the incidence of fatal, PE was 4 times higher than that of fatal bleeding (76 vs. 17 deaths, respectively). In contrast, in COPD patients initially presenting with DVT there were 17 PE recurrences and 31 major bleeding events, but the incidences of fatal recurrent PE and fatal bleeding were similar: 6 vs. 8 deaths. Thus, these observations suggest that in COPD initially presenting with PE the major concern should be recurrent (and potentially fatal) PE.

The main limitation in this study may be that the diagnosis of COPD in the RIETE registry may be questionable. No functional tests were systematically performed to diagnose COPD. This is unfortunately shared with the vast majority of other observational studies [25-28]. For example, only 28% of patients included in a recent study [28] of acute exacerbation of COPD had functional data. However, there is little chance that patients have been misclassified regarding their COPD status. The prevalence of COPD in the RIETE registry is similar to prevalence of COPD in general setting [29]. Moreover, COPD is usually underdiagnosed [30], and so, if misclassification exists, it should preferably be underdiagnosed. Nevertheless, the record of the presence of COPD was not different between PE and DVT patients, then not leading to any measurement bias. Furthermore, it could have been interesting to check whether the severity of COPD is associated with the initial VTE clinical presentation and whether it affects the association between PE presentation and the 3-month outcomes. Unfortunately, this variable was not available in the RIETE registry.

## Conclusion

In conclusion, we found a significant higher risk of recurrent VTE as PE and fatal PE in COPD patients presenting with PE compared to those presenting with DVT. As PE is the main cause of death during the 3-month follow-up, there is a huge need for treatment which would be more efficient in terms of PE occurrence but also which would not induce an increase in the bleeding risk, as there is also a trend in a increased risk of bleeding. In this view, retrievable vena cava filter [31], which protect the pulmonary vascular bed without increasing the bleeding risk, deserves further evaluations.

## Abbreviations

CI: Confidence interval; COPD: Chronic obstructive pulmonary disease; DVT: Deep-vein thrombosis; OR: Odds ratio; PE: Pulmonary embolism; VTE: Venous thromboembolism.

## Competing interests

The authors declare that they have no competing interests.

## Authors’ contributions

LB designed the study. LB, LH, JJMV, ARM, MM and the RIETE members included patients. SQ performed the statistical analyses. LB, SQ, and SL drafted the manuscript. All authors read and approved the manuscript.

## Authors’ information

Coordinator of the RIETE Registry: Dr. Manuel Monreal (Spain)

RIETE Steering Committee Members: Dr. Hervé Decousus (France), Dr. Paolo Prandoni (Italy), Dr. Benjamin Brenner (Israel)

RIETE National Coordinators: Dr. Raquel Barba (Spain), Dr. Pierpaolo Di Micco (Italy), Dr. Laurent Bertoletti (France), Dr. Manolis Papadakis (Greece), Dr. Marijan Bosevski (Republic of Macedonia), Dr. Henri Bounameaux (Switzerland), Dr. Radovan Malý (Czech Republic)

RIETE Registry Coordinating Center: S & H Medical Science Service

Members of the RIETE Group

SPAIN: Arcelus JI, Arcos MP, Ballaz A, Barba R, Barrón M, Barrón-Andrés B, Blanco-Molina A, Bosco J, Chaves E, Cañas I, Casado I, Contra A, Conget F, de Miguel J, del Campo R, del Toro J, Falgá C, Fernández-Capitán C, Gabriel F, Gallego P, García-Bragado F, Gavín O, Gómez V, González J, Gracia V, Guil M, Guillem N, Gutiérrez J, Hernández L, Hernández-Huerta D, Jaras MJ, Jiménez D, Jiménez S, Jiménez-Gil M, Lobo JL, Lecumberri R, López-Jiménez L, Lorenzo A, Macià M, Madridano O, Marchena PJ, Martín M, Martín-Villasclaras JJ, Monreal M, Morales M, Morán LP, Nauffal MD, Nieto JA, Núñez MJ, Mascareño MC, Ogea JL, Otero R, Pedrajas JM, Riera-Mestre A, Rodríguez-Dávila MA, Román P, Román-Bernal B, Roldán V, Rosa V, Royo C, Ruíz J, Ruiz-Gamietea A, Ruiz-Giménez N, Sahuquillo JC, Sánchez R, Sánchez Muñoz-Torrero JF, Soler S, Soto MJ, Tiberio G, Tolosa C, Trujillo J, Uresandi F, Valdés M, Valle R, Vela J, Vidal G, Villalta J, Zorrilla V; FRANCE: Bertoletti L, Bura-Riviere A, Debourdeau P, Farge-Bancel D, Lamuraglia M, Mahe I, Merah A, Quere I; GREECE: Babalis D, Papadakis M; ISRAEL: Brenner B; ITALY: Barillari G, Ciammaichella M, Di Micco P, Dalla Valle F, Duce R, La Regina M, Maida R, Orlandini F, Pasca S, Piovella C, Poggio R, Prandoni P, Quintavalla R, Rota L, Schenone A, Tonello D, Visonà A, Zalunardo B; REPUBLIC OF MACEDONIA: Bosevski M. SWITZERLAND: Bounameaux H; CZECH REPUBLIC: Malý R, Hirmerova J; ECUADOR: Salgado E.
